# Gender Differences in the Level of Antibodies to Measles Virus in Adults

**DOI:** 10.3390/vaccines9050494

**Published:** 2021-05-12

**Authors:** P. Mikhail Kostinov, I. Pavel Zhuravlev, N. Nikolay Filatov, M. Аristitsa Kostinova, B. Valentina Polishchuk, D. Anna Shmitko, V. Cyrill Mashilov, E. Anna Vlasenko, A. Alexey Ryzhov, M. Аnton Kostinov

**Affiliations:** 1 Federal State Budgetary Scientific Institution I.I. Mechnikov Research Institute of Vaccines and Sera, Maliyi Kazenniy Pereulok, 5a, 105064 Moscow, Russia; monolit.96@mail.ru (P.M.K.); pvazhurik@gmail.com (I.P.Z.); n.n.filatov@yandex.ru (N.N.F.); polischook@mail.ru (B.V.P.); violadellanna@gmail.com (D.A.S.); k.v.mashilov@gmail.com (V.C.M.); vaccinums@gmail.com (A.A.R.); mono469@gmail.com (M.A.K.); 2 Federal State Autonomous Educational Institution of Higher Education I.M. Sechenov First Moscow State Medical University of the Ministry of Health of the Russian Federation, Sechenov University, Trubetskaya Str., 8/2, 119991 Moscow, Russia; 3 National Research Center Institute of Immunology Federal Medical-Biological Agency of Russia, Kashirskoe Shosse, 24, 115478 Moscow, Russia; 4 Novokuznetsk State Institute for Advanced Training of Physicians, Branch Campus of the Russian Medical Academy of Continuous Professional Education, Prospect Stroiteley, 5, 654005 Novokuznetsk, Russia; VlasenkoAnna@inbox.ru

**Keywords:** measles, measles immunity among men and women, age characteristics of measles immunity

## Abstract

Individuals without a protective antibody level are susceptible to measles infection. There are differences in the persistence of antibodies after vaccination and infection, while the impact of gender on this process has not been sufficiently studied. Measles Ig G antibodies were measured in 1742 employees of a large hospital facility—403 men and 1339 women aged from 25 to 67 years; 15% participants had antibody levels less than the protective threshold of ≥0.18 IU/mL. Significant differences were found in the age group 40–49, where the level of IgG antibodies to measles among men was higher than among women (1.51 IU/mL (0.41; 3.38) vs. 0.70 IU/mL (0.22;1.98) respectively, (U = 3.2, *p* = 0,001)); in the age group 60 and older, by contrast, the level of antibodies among women was higher compared to men (3.29 IU/mL (1.72; 4.07) vs. 2.90 IU/mL (1.46; 3.53) respectively (U = 2.2, *p* = 0.03)). The proportion of seronegative women in the age group 40–49 was significantly higher than of seronegative men: 22 [18–26]% and 11 [6–18]% respectively (χ^2^ = 7.0, *p* = 0.001). The revealed gender characteristics that affect persistence of measles immunity may be important in personalization of vaccinal prevention for men and women.

## 1. Introduction

Before active universal vaccination, the incidence of measles was very high and ranked first among airborne infections among children of early age. Mass immunization has led to a sharp decline in the incidence, lack of periodicity, and seasonality [1,2]. At present, measles has ceased to be a «child» infection, and among the patients with measles the proportion of older children, adolescents, and adults has increased [3,4,5,6,7,8]. Numerous studies have proved that measles in adults has its own characteristics, which are not known to all practitioners, the consequence of which is late isolation and hospitalization of patients and untimely initiation of treatment with the subsequent development of severe complications.

From the published data, it is known that there is an existing gender difference in the formation of post-vaccinal immunity for administration of some vaccines. For example, after vaccination against tick-borne encephalitis, a significantly more expressed immune response in the dynamics of virus-neutralizing antibodies to virus strains accumulation was revealed among women of all age groups in comparison with men [9]. Accordingly, among men, a loss of protective antibody levels is observed in a shorter time period and high susceptibility to tick-borne encephalitis virus remains, and infection occurs more often compared to women [10]. However, the question of whether there are differences among the male and female population in the measles incidence rate, the disease severity, and the presence of immunity to this infection, is still open. In the available publications we did not find any information about the researches devoted to this problem. Probably the detection of such differences can contribute to the improvement of vaccination tactics in order to increase the immune layer for this infection, and men or women, depending on the result, can be attributed to infection risk groups equally with other categories of patients with health abnormalities [11,12,13,14,15].

The aim of the study was to evaluate the intensity of humoral immunity to measles among men and women working in a large hospital complex.

## 2. Materials and Methods

A single-step analytical study was carried out as part of a program to improve the work on measles prevention conducted in the entities of the Russian Federation, with an analysis of the reasons for growth of the proportion of seronegative people and comparison of the results of seromonitoring with data of age-related measles incidence [16].

### 2.1. Historical Information

In the USSR and then in the Russian Federation, mass vaccination of the population with one dose of the vaccine against measles was started in 1968, and since 1987, revaccination has been carried out with the administration of a booster dose. Currently, according to the national immunization schedule of the Russian Federation, measles vaccination is carried out for children aged 12–15 months with mandatory revaccination at 6–6.5 years. Also, immunization is recommended for all people under 35 years who have not suffered from measles, have not been vaccinated at all, or have been vaccinated once and who have no information about previous vaccinations against measles; in the case of an unfavorable epidemiological situation that has been observed in recent years, people under 55 years of age are vaccinated.

### 2.2. Contingent

After obtaining signed informed consent, the levels of IgG antibodies to measles were measured in 1742 employees (403 men and 1339 women aged 25 to 67 years) of a large hospital complex in Moscow in 2018. Respondents were divided by age and sex into four groups: up to 39 years old, 40–49 years old, 50–59 years old, and 60 years and older in agreement with the epidemiologist, due to the convenience of identifying groups with a difference of 10 years. Blood for study was taken during working hours in the morning in compliance with the antiseptic rules and ethical standards. No acute respiratory infections or exacerbations of concomitant diseases were reported among the staff at the time of examination.

Medical records of each employee contained information confirming vaccination against measles performed in the past, since this is a prerequisite condition for working in a medical facility.

The inclusion criteria were:

1. known vaccination history;
2. presence of medical documents on vaccination and revaccination against measles;
3. only employees of the medical organization participated in the study.

Since the production of postvaccinal antibodies and the duration of their maintenance can be influenced by various factors and health conditions of respondents, non-inclusion criteria were:

4. immunosuppressive therapy, systemic use or inhalation of corticosteroids in high doses (over 800 µg of beclomethasone or equivalent per day), radiotherapy, cytotoxic drugs or nonsteroid anti-inflammatory drugs;
5. HIV infection (positive serological test), hepatitis B (acute form) and hepatitis C (acute form);
6. therapy containing immunoglobulins and other donor blood products within 90 days prior to the study participation;
7. any vaccine administration within 30 days prior to inclusion in the study;
8. contraindications to introduction of the measles vaccine (according to the instruction);
9. the accession of acute respiratory infections during the first days after vaccination.

### 2.3. Vaccines

For measles prevention in the Russian Federation, both mono- and combined with the antigen of mumps home-produced vaccines are used. The monoprepararion is a live measles cultural vaccine prepared from the vaccine strain L-16 (Leningrad-16) or its cloned variant-Moscow-5 grown on the culture of Japanese quail embrio cells (FSUE “Scientific and Production Association for Immunological Preparations NPO “Microgen”). Since 2001, associated mumps-measles vaccine has been used, which is produced in the same way as monovalent vaccine [17].

Among foreign preparations, Priorix (GlaxoSmithKlein, Binford, UK (Belgium) and MMR II (Merck, Sharp & Dohme, Fort kennarworth, NJ, USA)) were registered in Russia, which were rarely used and mostly in private clinical practice. Comparative data of the study of safety and immunogenicity of domestic and foreign measles vaccines did not reveal significant differences [13,18,19].

### 2.4. Laboratory Methods

Measles virus IgG antibodies were measured by enzyme-linked immunosorbent assay (ELISA), using kit «Vector-best IgG–measles» (Russia) in a licensed laboratory. According to enclosed regulatory and technical documentation for the quantitation of IgG antibodies to measles virus, the result of the analysis was considered negative if antibodies concentration in the sample was less than 0.12 IU/mL; it was positive if the concentration was equal or more than 0.18 IU/mL. Samples with doubtful concentrations of IgG antibodies to measles virus (in the range 0.12–0.17 IU/mL) were interpreted as negative, because this level of antibodies cannot be considered significantly protective.

### 2.5. Statistical Analysis

A preliminary analysis of the data showed that the distribution of IgG antibodies to measles virus was different from normal among both men and women (W = 0.87, *p* < 0.001 and W = 0.88, *p* < 0.001, respectively). The Box-Tidwell linearity test revealed that the relationship between age and IgG levels (taking into account the respondent’s sex) was statistically significantly different from linear (z = 1.96, *p* = 0.05). Therefore, the local polynomial regression (LOESS regression) with the calculation of 95% confidence interval was used for the regression analysis; the assessment was carried out using the bootstrap method based on 9999 cycles (spatialEco v.1.2–0 package). In order to compare the level of antibodies depending on the sex of the respondents, Mann-Whitney test was applied. A comparison of the level of antibodies between age groups within the same sex was carried out by the Mann-Whitney test with Holm correction. A comparison of the proportion of seronegative respondents depending on sex was carried out by the Chi-Square criterion, depending on the age group within one sex, by the Chi-Square criterion with Holm correction. In the case of cells with expected frequencies less than 5%, Fisher’s exact test was applied. If there were statistically significant differences, the relative risk and its 95% confidence interval was calculated. Descriptive statistics of quantitative data were represented by median and interquartile ranges of the quantitative proportions of respondents with considered characteristics in the group, with an indication of 95% confidence interval calculated by the Clopper-Pearson method. The absolute number of respondents with a trait from the total group size (n N) was also indicated. All calculations were performed in the free computing environment R (v.3.6.0).

## 3. Results

The analysis of the laboratory results of the study in which 1742 people aged 25–67 years (403 men and 1339 women) were involved, showed that the median of IgG antibodies level to measles virus in samples was 1.2 (0.4–3.2) IU/ mL; the proportion of seronegative respondents was 15 [14–17]%. Without taking into account the age of the respondents between men and women, no statistically significant differences were found neither in the level of IgG antibodies (1.07 (0.4; 3.0) IU/mL in the group of men and 1.3 (0.4; 3.3) IU/mL in the group of women (U = 1.15, *p* = 0.28)), nor in the part of seronegative respondents (13 [10–17]% (54/403) among men and 16 [14–18]% (214/1339) among women (χ^2^ = 1.6, *p* = 0.21)).

**Figure 1:**
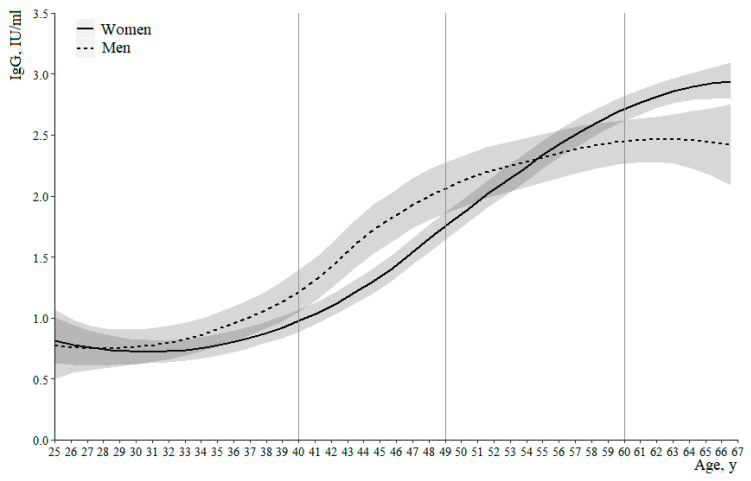
The results of approximation (LOESS-regression) of the relationship of IgG-antibody levels to measles virus and the age of respondents depending on sex.

An approximation of the relationship between the respondents’ age and the IgG antibody levels to measles virus was performed by local polynomial regression (LOESS) with the use of bootstrap; the analysis was carried out separately for men and women (Table 1, Figure 1).

**Figure 2:**
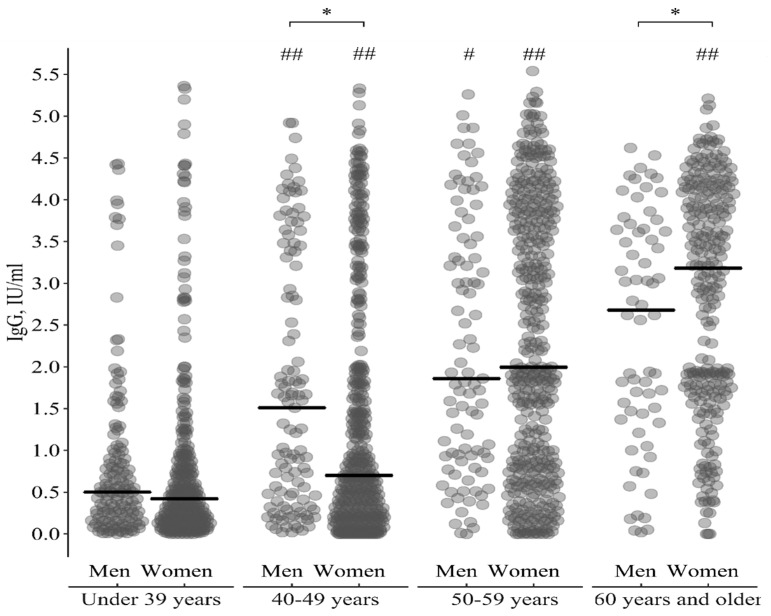
Individual values and median of IgG antibodies levels to measles virus in selected age groups depending on sex. Note: *—statistically significant differences between men and women at level *p* ≤ 0.05, #—statistically significant differences compared to the previous age period at level *p* ≤ 0.05, ##—statistically significant differences compared to the previous age period at level *p* ≤ 0.001.

From Figure 1 and Table 1 it can be seen that for some age groups the confidence intervals of regression lines do not intersect (aged groups 40–49 and 60 and older). This indicates possible statistically significant differences between men and women in the level of IgG antibodies to measles virus in these age intervals. Therefore, taking into account the intersection of the confidence intervals of the regression lines, the whole age range can be divided into the following four groups: under 39 years, 40–49 years, 50–59 years, and 60 years and older. The levels of the anti-measles IgG antibodies in the selected age groups depending on sex are presented in Table 2 and Figure 2.

At the age of 40–49 years old, the level of IgG antibodies to measles virus among men is statistically significantly higher than among women; and at the age of 60 years and older, on the contrary, the level of antibodies is higher among women in comparison with men. Antibody levels increase with age in both men and women. However, if among men after 50 years old the growth rate slows down, at the age of 60 years and older compared to the age group of 50–59 years it does not change, then among women an increase in antibodies is observed in each age period.

**Figure 3:**
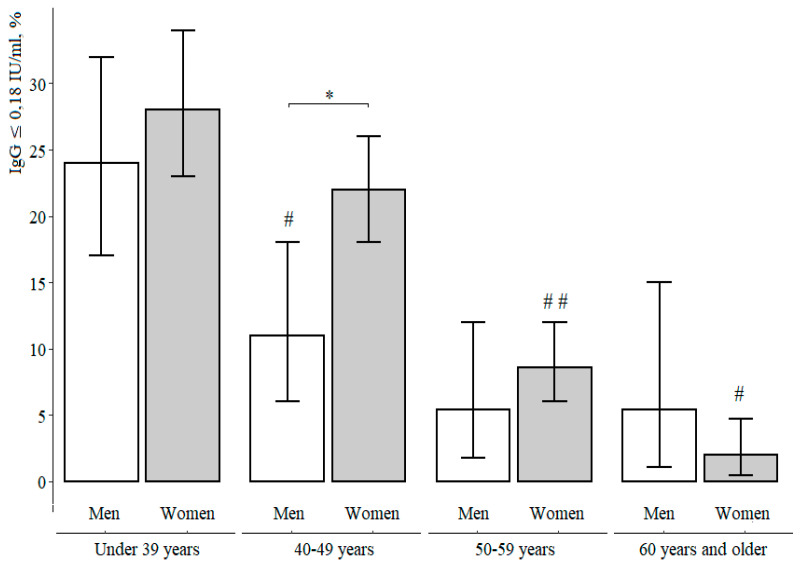
The proportion of seronegative (IgG < 0.18 IU/mL) respondents in the selected age groups depending on sex. Note: *—statistically significant differences between men and women at level *p* ≤ 0.05, #—statistically significant differences compared to the previous age period at level *p* ≤ 0.05, # #—statistically significant differences compared to the previous age period at level *p* ≤ 0.001.

Furthermore, we consider the proportion of seronegative respondents depending on sex in each selected age group (Table 3, Figure 3.)

In the age group 40–49 years old, the proportion of seronegative women is statistically significantly higher than the proportion of seronegative men: 22 [18–26]% and 11 [6–18]%, respectively (χ^2^ = 7.0, *p* = 0.001).

It should also be noted that a statistically significant decrease in the proportion of seronegative respondents among men is observed after the age of 40 years (with further stabilization) and among women after the age of 50 years (with a further decrease). Despite the differences in dynamics, the proportion of seronegative respondents among men and women over the age of 60 did not differ statistically: 5.4 [1.1–15]% and 2.0 [0.5–4.7]%, respectively (*p* = 0.16 according to Fisher).

## 4. Discussion

There are peculiarities in the formation and maintenance of humoral immunity to measles infection, which depend on the state of health of the vaccinated person, and on the scheme and the number of vaccine administrations. For example, when vaccinating patients with allergic diseases, delayed synthesis of measles antibodies is noted, and the terms of their preservation in protective values are reduced [20,21]. In order to intensify the production of specific antibodies, vaccination is accompanied by the administration of one of the immunocorrective drugs that leads to activation and regulation of the immune response mechanism, including the reduction of cases of respiratory infections in the postvaccinal period in the cold season, which can lead to impaired formation of an adequate immune response [22,23,24,25]. Patients with autoimmune diseases, HIV-infected people, and people who receive immunosuppressive therapy also have a rapid loss of postvaccinal immunity, and this is the reason why constant monitoring of the level of specific antibodies with the introduction of booster doses in the absence of their protective values is recommended [26]. In our study, according to the criteria of non-inclusion, respondents did not have diseases that could affect the immunity to measles virus.

It should be noted that the two-fold administration of the vaccine regardless of the state of the vaccinated person is accompanied by the synthesis of antibodies to the measles virus in higher values, which persist for a longer time in comparison with a single administration. This vaccination scheme that leads to the formation of the immune layer in the population and the creation of a favorable epidemic situation with respect to measles, is currently used in all countries. According to the available information reflected in the medical card, each employee of the medical institution included in the study had information about the vaccination carried out in accordance with the age.

It is known that after measles, antibodies are able to protect the person from subsequent infection. In the blood of adult patients with measles who were not vaccinated in childhood, there are high levels of specific IgM and IgA, and an increase in the number of IgG antibodies with low avidity, mainly of the IgG3 subclass. At the same time for previously vaccinated patients, low IgM concentration and high concentrations of specific IgA and IgG with high avidity, mainly IgG1-subclass, is typical. The humoral immune response in people who have been vaccinated within the prescribed terms is characterized by a lower concentration of measles-specific IgA, as well as IgG and avidity, than in those who have had measles in childhood. The association of some HLA haplotypes with the intensity of immune response to measles has been revealed [27]. In addition, there are gender differences in postvaccinal period after the administration of measles, mumps, and rubella vaccines. Women are more likely to develop adverse events such as fever, lymphadenitis, and manifestation of parotitis in response to vaccination [28], with the exception of immune thrombocytopenic purpura, which is more common among men [29]. The study did not include respondents with confirmed information about measles in the past history and/or with the development of unusual events in the postvaccinal period.

Therefore, there are differences in the course of the postvaccinal period and in the mechanisms of antibody formation to the measles virus and their persistence, which can affect the epidemic process as a whole.

An increase in the number of outbreaks of measles infection, including in medical institutions, to which attention is always drawn because of the availability of vaccination against this disease, led us to conduct this study. It was revealed that among the employees of the hospital complex aged 25 and 67, 15% of subjects had antibody levels of less than the protective threshold. A relatively large proportion of seronegative individuals does not fit into the conditionally safe range to create a favorable epidemic situation (presence of seronegative people about 7%) and the risk of measles outbreaks remains. However, it is interesting that there are differences in the levels of protective antibodies among men and women; the latter being more likely to work in health facilities (1339 women and 403 men in our study). In the analysis of sex differences on the detection of the level of IgG antibodies and the proportions of seronegative employees without taking into account the age of respondents, there were no statistically significant differences between men and women. However, in the distribution of health workers by age groups, there were differences in the levels of IgG antibodies to the measles virus: Among men aged 40–49, the level was statistically significantly higher than among women. Consequently, the relative risk of lack of protective IgG levels among women aged 40–49 is 2 [1–3.6] times higher than among men. At the age of 60 and older, in contrast, the level of antibodies is higher among women in comparison with men. It should be noted that with an age of 60 years and older, the proportion of people with low antibody levels decreases and the proportion of people with average levels of antibodies to measles virus increases among both sexes [30]. Of course, the revealed fact may be associated with the measles disease before the era of measles vaccination, although vaccination of people who have had atypical forms of the disease cannot be ruled out.

As for the proportion of seronegative to the measles virus persons, they are more often detected among women of the age group 40–49, making up 22%, compared to 11% in men of the same age (χ^2^ = 7.0, *p* = 0.001).

Decrease in the measles-specific antibody levels and registration of a large proportion of seronegative to measles virus among young men and women under 39 years old (24 [17–32]% and 28 [23–34]%, respectively) does not mean a complete loss of measles immunity, since the response of memory B-cells can quickly increase the synthesis of protective antibodies [27]; however, from our point of view, this does not exclude the patient’s susceptibility to measles with the development of the clinical presentation of the disease.

Of interest are the results of the works of a number of authors, which are devoted to the study of sex differences in the formation of the immune response after vaccination against vaccine-preventable infections. Existing regression models show that the two most significant predictors of antibody production and effectiveness of vaccination are age at the time of vaccination and female sex [31]. For example, in a study conducted in 2006, it was shown that in response to the introduction of rubella, measles, and mumps vaccines in children 12–15 months after birth, serum IgG production against the main components of the vaccine was significantly higher among girls in comparison with boys [32]. It has also been shown that among girls aged 14–17 years, higher IgG titers against rubella virus are recorded [33]. Our study on the level of antibodies among employees of a large hospital facility found sex differences in the age groups of 40–49 years and 60 years and older, in which the levels of IgG antibodies to the measles virus were found statistically significantly higher at the beginning among men and then among women.

Thus, it can be assumed that sex differences of immune response can contribute to the difference in the pathogenesis of infectious diseases among men and women, and in the development of reactions to vaccine preparations. Sex differences in response to vaccination are observed among different age groups, ranging from infants to the elderly. In some articles, a higher level of post-vaccination antibodies to vaccine-preventable infections in women is associated not only with sex steroids (associated with estradiol) throughout life, but also with the expression of a X-linked gene located on the X chromosome, Tlr7» [34,35,36]. Biological as well as behavioral differences between the sexes also probably contribute to the formation of different variants of the course of the postvaccinal period. For example, women are characterized by the development of a more pronounced specific immune response, as well as adverse events on vaccine administration. On the other hand, women are more susceptible to autoimmune diseases. Knowledge of the mechanisms involved in the sex inequality of the immune response can help to identify ways to reduce the development of adverse events in the postvaccinal period among women and to improve immune response on vaccine administration among men [19,22,37]. This is necessary for adequate protection of both sexes from infectious diseases in order to personalize vaccinal prevention of men and women [38,39,40,41].

## 5. Conclusions

The study revealed that due to the start of mass vaccination against measles of the population of the Russian Federation in 1968, nowadays specific immunity at the protective level in adults, in particular among employees of a large hospital complex (aged 25–67), is detected in 85% of cases regardless of gender, and the lack of protection to the measles virus is noted in 15% of employees. However, among women aged 40–49, IgG antibody levels are recorded in values lower, and the proportion of seronegative to the measles virus is higher than among men. Significant differences are observed in the age group 60 years and older, in which, on the contrary, the level of IgG antibodies is higher among women in comparison with men. Despite the duration of preservation of protective antibodies, men have revealed a pronounced dynamics of their decline, regardless of the nature of their formation—vaccination or infection (before the era of vaccination 1968).

## Figures and Tables

**Table 1:** The results of approximation (LOESS-regression) of the relationship between the levels of IgG-antibodies to measles virus (IU/mL) and the age of respondents depending on sex.

Age	Regression Assessment of IgG Levels and 95% Confidence Interval		Age	Regression Assessment of IgG Levels and 95% Confidence Interval	
	Women	Men		Women	Men
25	0.81 [1.00–0.63]	0.78 [108–0.51]	47	1.61 [1.72–1.50]	2.00 [2.19–1.79]
26	0.78 [0.95–0.62]	0.76 [1.00–0.56]	48	1.71 [1.,82–1.60]	2.06 [2.26–1.86]
27	0.76 [0.90–0.62]	0.75 [0.95–0.57]	49	1.81 [1.92–1.70]	2.11 [2.31–1.92]
28	0.74 [0.86–0.62]	0.75 [0.92–0.59]	50	1.91 [2.02–1.80]	2.16 [2.35–1.95]
29	0.73 [0.84–0.63]	0.75 [0.91–0.61]	51	2.01 [2.13–1.90]	2.19 [2.39–1.98]
30	0.72 [0.82–0.63]	0.76 [0.91–0.63]	52	2.11 [2.23–2.00]	2.22 [2.43–2.01]
31	0.72 [0.81–0.64]	0.78 [0.92–0.65]	53	2.20 [2.33–2.10]	2.26 [2.47–2.05]
32	0.73 [0.82–0.65]	0.80 [0.94–0.67]	54	2.30 [2.42–2.20]	2.30 [2.51–2.10]
33	0.74 [0.83–0.66]	0.83 [0.97–0.71]	55	2.39 [2.50–2.29]	2.34 [2.54–2.14]
34	0.76 [0.85–0.67]	0.87 [1.01–0.74]	56	2.48 [2.59–2.37]	2.37 [2.57–2.17]
35	0.78 [0.87–0.70]	0.91 [1.05–0.78]	57	2.56 [2.67–2.46]	2.40 [2.59–2.21]
36	0.81 [0.90–0.72]	0.96 [1.11–0.82]	58	2.64 [2.75–2.54]	2.42 [2.60–2.22]
37	0.85 [0.94–0.76]	1.01 [1.17–0.87]	59	2.72 [2.82–2.58]	2.43 [2.61–2.25]
38	0.89 [0.98–0.80]	1.07 [1.24–0.93]	60	2.80 [2.90–2.65]	2.44 [2.62–2.28]
39	0.94 [1.03–0.84]	1.15 [1.32–1.00]	61	2.80 [2.91–2.69]	2.46 [2.63–2.29]
40	0.99 [1.08–0.90]	1.25 [1.42–1.08]	62	2.81 [2.92–2.72]	2.47 [2.64–2.29]
41	1.06 [1.14–0.96]	1.37 [1.55–1.19]	63	2.86 [2.97–2.76]	2.46 [2.66–2.27]
42	1.13 [1.22–1.03]	1.50 [1.68–1.29]	64	2.90 [3.01–2.79]	2.46 [2.69–2.24]
43	1.20 [1.31–1.10]	1.62 [1.80–1.40]	65	2.92 [3.05–2.80]	2.44 [2.73–2.18]
44	1.29 [1.39–1.18]	1.72 [1.92–1.51]	66	2.94 [3.09–2.79]	2.42 [2.74–2.11]
45	1.39 [1.49–1.28]	1.82 [2.01–1.61]	67	2.95 [3.14–2,.77]	2.41 [2.76–2.02]
46	1.50 [1.61–1.39]	1.91 [2.11–1.71]			

**Table 2:** Median and interquartile range of IgG antibodies levels to measles virus in the selected age groups depending on sex.

Age Groups	IgG, IU/Ml—Me(IQR)		Comparison by Sex
	Men	Women	
Under 39 y	0.50(0.19; 0.97)	0.42(0.16; 0.91)	U = 1.11, *p* = 0.27
40–49 y	1.51(0.41; 3.38)	0.70(0.22; 1.98)	U = 3.2, *p* = 0.001
50–59 y	1.86(0.93; 3.54)	2.00(0.79; 3.83)	U = 0.29, *p* = 0.77
60 y and older	2.68(1.46; 3.53)	3.29(1.72; 4.07)	U = 2.2, *p* = 0.03
Comparison of age groups in dynamics:			
40–49 y/under 39 y	U = 5.2, *p*_h_ < 0.001	U = 4.7, *p*_h_ < 0.001	-
50–59 y/40–49 y	U = 2.3, *p*_h_ = 0.04	U = 9.0, *p*_h_ < 0.001	
60 y and older/50–59 y	U = 1.3, *p*_h_ = 0.19	U = 4.4, *p*_h_ < 0.001	

**Table 3:** The proportion of seronegative respondents (IgG < 0.18 ME/mL) in selected age groups depending on sex.

Age Groups		IgG < 0.18 IU/mL		Comparison by Sex
		Men	Women	
Under 39 y	% [CI]	24 [17–32]%	28 [23–34]%	χ^2^ = 0.87, *p* = 0.27
	n/N	34/141	85/300	
40–49 y	% [CI]	11 [6–18]%	22 [18–26]%	χ^2^ = 7.0, *p* = 0.001
	n/N	12/113	90/415	
50–59 y	% [CI]	5.4 [1.8–12]%	8.6 [6.0–12]%	χ^2^ = 1.1, *p* = 0.77
	n/N	5/93	35/408	
60 y and older	% [CI]	5.4 [1.1–15]%	2.0 [0.5–4.7]%	*p* = 0.16 according to Fisher
	n/N	3/56	4/216	
Comparison of age groups in dynamics				
40–49 y/under 39 y		χ^2^ = 7.7, *p*_h_ = 0.02	χ^2^ = 3.8, *p*_h_ = 0.06	-
50–59 y/40–49 y		χ^2^ = 1.9, *p*_h_ = 0.35	χ^2^ = 24.4, *p*_h_ < 0.001	
60 y and older/50–59 y		χ^2^ = 0.01, *p*_h_ = 0.99	χ^2^ = 10.9, *p*_h_ = 0.001	
